# A systematic review of cytokines in chronic fatigue syndrome/myalgic encephalomyelitis/systemic exertion intolerance disease (CFS/ME/SEID)

**DOI:** 10.1186/s12883-019-1433-0

**Published:** 2019-08-24

**Authors:** Matthew Corbitt, Natalie Eaton-Fitch, Donald Staines, Hélène Cabanas, Sonya Marshall-Gradisnik

**Affiliations:** 1 National Centre for Neuroimmunology and Emerging Diseases, Menzies Health Institute, Griffith University, Gold Coast, Australia; 2 School of Medical Science, Griffith University, Gold Coast, Australia; 3 Consortium Health International for Myalgic Encephalomyelitis, National Centre for Neuroimmunology and Emerging Diseases, Griffith University, Gold Coast, QLD Australia

**Keywords:** Chronic fatigue syndrome, Myalgic encephalomyelitis, Systemic exertion intolerance disease, Cytokines

## Abstract

**Background:**

Cytokines in Chronic Fatigue Syndrome/Myalgic Encephalomyelitis/Systemic Exertion Intolerance Disease (CFS/ME/SEID) patients compared with healthy controls have been extensively studied. However, the evidence regarding whether a baseline difference between CFS/ME/SEID patients and the normal population remains unclear. The aim of this study was to conduct a systematic review of the literature regarding cytokines in CFS/ME/SEID and whether there is a significant difference in cytokine levels between this patient group and the normal population.

**Methods:**

Pubmed, Scopus, Medline (EBSCOHost), and EMBASE databases were searched to source relevant studies for CFS/ME/SEID. The review included any studies examining cytokines in CFS/ME/SEID patients compared with healthy controls. Results of the literature search were summarised according to aspects of their study design and outcome measures, namely, cytokines. Quality assessment was also completed to summarise the level of evidence available.

**Results:**

A total of 16,702 publications were returned using our search terms. After screening of papers according to our inclusion and exclusion criteria, 15 studies were included in the review. All the included studies were observational case control studies. Ten of the studies identified measured serum cytokines in CFS/ME/SEID patients, and four measured cytokines in other physiological fluids of CFS/ME/SEID patients. The overall quality assessment revealed most papers included in this systematic review to be consistent.

**Conclusions:**

Despite the availability of moderate quality studies, the findings of this review are inconclusive as to whether cytokines play any definitive role in CFS/ME/SEID, and consequently, they would not serve as reliable biomarkers. Therefore, in light of these results, it is recommended that further efforts toward a diagnostic test and treatment for CFS/ME/SEID continue to be developed in a range of research fields.

**Electronic supplementary material:**

The online version of this article (10.1186/s12883-019-1433-0) contains supplementary material, which is available to authorized users.

## Background

Chronic Fatigue Syndrome, Myalgic Encephalomyelitis or Systemic Exertion Intolerance Disease (CFS/ME/SEID), is a complex, chronic illness with an unknown aetiology that causes a significant reduction in a person’s quality of life [1, 2]. It is characterised by a relapsing-remitting pattern of unexplained fatigue and alterations in memory and concentration, amongst other debilitating symptoms [3, 4]. Globally, the prevalence of CFS/ME/SEID is between 0.8 and 3.3% [5], with associated costs in the United States being up to USD10,000 per patient [6], USD7 billion directly to society [6] and USD9.1 billion in productivity losses [7]. In conjunction with its undetermined aetiology and costly clinical management, other challenging aspects of CFS/ME/SEID include the lack of a standardised diagnostic test, as diagnosis is still dependent upon specific clinical criteria, and no available targeted treatments [3, 8].

Currently, the Fukuda (1994) criteria [8] is the most commonly applied case definition [1]. The criteria specify that for patients to be diagnosed with CFS/ME/SEID, they must have debilitating fatigue for at least 6 months, which interferes with daily activities, and the cause cannot be explained by excessive exertion, or other psychiatric or medical conditions. Additionally, a further four or more of the following symptoms must also be present: post-exertional malaise, difficulty with short-term memory or concentration, unrefreshing sleep, sore throat, muscle pain, joint pain, tender lymph nodes, and headaches [8]. However, more recently the International Consensus Criteria (ICC) has been able to provide an alternative set of criteria to Fukuda (1994), and has allowed for further recognition of variable symptomatology in CFS/ME/SEID patients [3]. Other criteria, such as the Canadian Consensus Criteria (CCC) [9] also exist, further highlighting the variability in diagnosis, and therefore, the need for a diagnostic test.

Limited understanding in the pathogenesis of CFS/ME/SEID not only makes the development of a diagnostic test difficult, it also essentially makes the production of targeted treatments challenging, and many conventional and alternative methods have limited evidence for their use in treatment of CFS/ME/SEID [1, 2, 10–13]. Nevertheless, a number of varying mechanisms have been proposed, including immunological dysfunction. Although evidence for immunologic abnormalities in CFS/ME/SEID has existed for nearly three decades [14], additional studies have proposed imbalances in pro-inflammatory and anti-inflammatory cytokines as a major contributor to the pathogenesis of CFS/ME/SEID [15–17] and a number of more recent studies have attempted to further characterise this.

Cytokines, by definition, are small, secreted proteins that are released by cells and play an important role in cellular signalling. They are especially important in inflammation and mediating the immune response and evidence exists for cytokines playing a role in chronic pain [18]. They can be further subcategorised into pro-inflammatory (e.g. IL-1β, IL-6, TNF-α) and anti-inflammatory (e.g. IL-4, IL-10, TGF-β) types, and via their opposing mechanisms, moderate the immune response. Additionally, chemokines (chemotactic cytokines) and interleukins (IL; lymphocyte-to-lymphocyte cytokines) are two important classes of cytokines that play a central role in the cytokine network as mediators of the immune response [18].

The understanding of CFS/ME/SEID’s complex aetiology is of critical importance for the development of a diagnostic test and effective treatment regimens, especially due its cost to both the patient and society. Despite some indication for cytokines playing an important role, the exact mechanism remains elusive. The focus of this review was exclusively targeted on cytokines in physiological fluids of CFS/ME/SEID patients, not immune cell cytokine production. Therefore, the aim of this study was to conduct a systematic review of the current literature regarding cytokines in CFS/ME/SEID to determine whether alterations exist when compared with healthy individuals.

## Methods

### Literature search

The study was performed according to PRISMA (Preferred Reporting Items for Systematic Reviews and Meta-Analyses) guidelines. Pubmed, Medline (EBSCOHost), Embase and Scopus were systematically searched, the primary search completed by the first author (MC) on April 28th 2018 and the secondary search completed by the second author (NE) on June 13th 2018. A final search was performed on June 27th 2018 as quality assurance to confirm no additional papers could be included. All searches did not identify new publications to be included in this review and employed the same method.

The following full-text terms were searched: ‘chronic fatigue syndrome’ OR ‘myalgic encephalomyelitis’ OR systemic exertion intolerance disease’ OR ‘CFS/ME/SEID’ AND ‘cytokines’. MeSH (medical Subject Headings) terms were used for ‘Syndrome, chronic fatigue’ (includes CFS/ME, chronic fatigue syndrome, myalgic encephalomyelitis, systemic exertion intolerance disease, SEID) and cytokines. Boolean operator ‘OR’ was used to combine all expressions of cases including abbreviations, while ‘AND’ was used to include cytokines in conjunction with CFS/ME/SEID in the search. Proximity operators were not used during the literature search. As stated above, two literature searches were completed for this systematic review on separate occasions by two authors and using the same method. Reference list checking and citation searching was completed, and no additional papers were selected. Searching for unpublished literature was not performed.

### Inclusion and exclusion criteria

Papers were screened according to the following criteria: (i) all studies reporting on cytokines in CFS/ME/SEID patients; (ii) studies that were published between 1994 and 2018 to exclude non-Fukuda based case definitions prior to 1994 [8]; (iii) human studies in adults aged 18 years and above; (iv) studies published in English; (v) free full text publications based upon original research; and, (vi) CFS/ME diagnosis according to Fukuda (1994) [8], Canadian (2003) [19] or International (ICC) (2011) [3]. Once screened, duplicates were subsequently removed. To control for possible publication bias, all full text publications were screened in addition to free publications, and one additional publication was included.

Studies were excluded if: only one out of three keywords were present in the title or abstract; the CFS/ME/SEID criteria was not defined by a single clinical criterion; the study was an interventional study; cytokine expression was induced in vitro; cytokines were not measured from a physiological fluid; where CFS/ME/SEID patients had other inflammatory or immune-based comorbidities; and, if the CFS/ME/SEID patient’s were not compared with healthy controls.

The primary outcome of this review was serum cytokine levels in CFS/ME/SEID patients. Secondary outcomes evaluated were cytokine levels in other body fluids of CFS/ME/SEID patients, such as cerebrospinal fluid (CSF) and nasal lavage. Studies were included on the provision that appropriate statistical analysis was performed to directly compare CFS/ME/SEID patients with healthy controls and this was outlined in their methodology. In addition, one study [15] meeting criteria for inclusion was excluded from this paper as it used a previously analysed patient group in another study [20] for its analysis, and therefore did not contribute any new data.

### Selection of studies and data extraction

The inclusion and exclusion criteria mentioned above was used for publication selection. After the review of abstracts and titles by two authors, full texts were also screened and underwent data collection. A summary of study characteristics, including: (i) author; (ii) year of publication; (iii) study design; (iv) patient diagnostic criteria; (v) cytokine origin; (vi) cytokine analysis method; (vii) country of research; and (viii) sample size, were extracted from each included publication and provided in Table 1. The vote counting approach was used to catalogue all information, as reported in all publications. This information was manually entered as tables and statistical significance was discussed in this systematic review. An overview of results as stated by each publication was outlined in Tables 2, 3 and 4, including cytokines assayed, outcomes, frequency of cytokine analysis and statistical significance defined by *p*-values. Included papers, whereby p-values were published as 0.000, were changed during data extraction to < 0.001. The methodology used in this systematic review was not registered with online registers of systematic reviews, such as PROSPERO, however the methodology used was adapted from previous publications [1, 2, 36].

**Table 1:** Summary of study characteristics of the included studies

Observational Studies							Sample Size	
Author	Year	Study Design	Dx	Cytokine Origin	Cytokine Analysis	Country	CFS/ME	HC
Fletcher, et al. [20]	2009	Case Control	Fukuda	Serum	ELISA	U.S.A.	40	59
Hardcastle, et al. [21]	2015	Case Control	Fukuda	Serum	MBAA	Australia	41	22
Hornig, et al. [22]	2016	Case Control	Fukuda and CCC	CSF	MBAA	U.S.A.	32	19
Kennedy, et al. [23]	2004	Case Control	Fukuda	Serum	ELISA	United Kingdom	47	34
Landi, et al. [24]	2016	Case Control	Fukuda and/or CCC	Serum	ELISA	U.S.A.	100	79
Montoya J, et al. [25]	2017	Case Control	Fukuda	Serum	MBAA	U.S.A.	186	388
Nakamura, et al. [26]	2010	Case Control	Fukuda	Serum	MBAA	U.S.A.	11	24
Nas K, et al. [27]	2011	Case Control	ICC	Serum	MBAA	Turkey	25	20
Natelson, et al. [28]	2005	Case Control	Fukuda	CSF	MBAA	U.S.A.	44	13
Neu D, et al. [29]	2014	Case Control	Fukuda	Serum	MBAA	Belgium	13	11
Peterson, et al. [30]	2015	Case Control	Fukuda	CSF	MBAA	U.S.A.	18	5
Repka-Ramirez, et al. [31]	2002	Case Control	Fukuda	Nasal Lavage	ELISA	U.S.A.	95	89
Russell, et al. [32]	2016	Case Control	ICC	Serum	ELISA	U.S.A.	50	69
Suhadolnik, et al. [33]	2004	Case Control	Fukuda	Serum	ELISA	U.S.A.	66	62
Tomoda et al. [34]	2005	Case Control	Fukuda	Serum	ELISA	Japan	15	23

*Dx* CFS/ME diagnostic criteria used, *HC* healthy control(s), *ELISA* enzyme-linked immunosorbent assay, *MBAA* multiplex bead array assay, *CCC* Canadian Consensus Criteria, *ICC* International Consensus Criteria, *CSF* cerebrospinal fluid

This table is a summary of the characteristics of the included studies

**Table 2:** Study results for the primary outcome of serum cytokine levels

		Serum Cytokine Levels	
Author	Cytokines Assayed	Increased in CFS/ME vs. HC (*p*-value)^a^	Decreased in CFS/ME vs. HC (p-value)^a^
Fletcher, et al. [20]	IL-Iα, IL-1β, IL-2, IL-4, IL-5, IL-6, IL-8, IL-10, IL-12p70, IL-13, IL-15, IL-17, IL-23, IFN- γ, LTα, TNF-α	LTα (0.000), IL-6 (0.000), IL-1α (0.044), IL-1β (0.041), IL-12 (0.000), IL-4 (0.000), IL-5 (0.000)	IL-8 (0.002), IL-13 (0.002), IL-15 (0.000)
Hardcastle, et al. [21]	IL-1β, IL-1RA, IL-2, IL-4, IL-5, IL-6, IL-7, IL-8, IL-9, IL-10, IL-12p70, IL-13, IL-17, FGF, CCL11, G-CSF, GM-CSF, IFN-γ, CXCL10, TNF-α, CCL2, CCL3, CCL4, CCL5, VEGF	IL-7 (< 0.001), IL-8 (0.001), CCL5 (0.009)	IL-6 (< 0.001)
Kennedy, et al. [23]	TGF-β1	TGF-β1 (0.005)	none
Landi, et al. [24]	CCL11, CCL24, CCL26, IL-8, CXCL10, CCL2, CCL13, CCL22, CCL3, CCL4, CCL17, GM-CSF, IL12/23p40, IL- 15, IL-16, IL-17A, IL-1a, IL-5, IL-7, TNF-β, VEGF-A, IFN-γ, IL-10, IL-12p70, IL-13, IL-1b, IL-2, IL-4, IL-6, TNF-α, CX3CL1, CXCL9, CCL19	CCL24 (0.007), CCL19 (0.04)	IL-16 (9.38 × 10^− 9^), IL-17A (2.79 × 10^− 5^), IL-7 (2.74 × 10^− 4^), TNF-β (0.003), CXCL9 (0.006), CX3CL1 (0.01), IL-1β (0.04), VEGF-A (0.01)
Montoya J, et al. [25]	CCL2, CCL3, CCL4, CCL5, CCL7, CCL11, CXCL1, CXCL5, CXCL9, CXCL10, FGF-basic, G-CSF, GM-CSF, HGF, IFN-α, IFN-β, IFN-γ, IL-1RA, IL-1α, IL-1β, IL-2, IL-4, IL-5, IL-6, IL-7, IL-8, IL-10, IL-12p40, IL-12p70, Il-13, IL-15, IL-17, IL-17F, LIF, M-CSF, Resistin, SCF, TGF-α, TGF-β, TNF-α, TNF-β, VEGF, Leptin	TGF-β (0.0052)	Resistin (0.0052)
Nakamura, et al. [26]	IL-1β, IL-4, IL-6, IL-8, IL-10, TNF-α	IL-10 (0.05)	none
Nas K, et al. [27]	IL-6, IL-8	IL-6 (0.007)	none
Neu D, et al. [29]	IFN-γ, IL-1β, IL-6, IL-8, IL-10, TNF-α	IL-1β (0.000), IL-8 (0.000), IL-10 (0.015), TNF-α (0.019)	IFN-γ (0.000), IL-6 (0.001)
Russell, et al. [32]	IL-1α, IL-1β, IL-2, IL-4, IL-5, IL-6, IL-8, 1 IL-10, IL-12p70, IL-13, IL-15, IL-17, IL-23, IFN-γ, TNF-α, TNF-β, LTα	IL-4 and IL-5 (*p* = 0.00 for >50yo), IL-12p70 (*p* = 0.02 for >50yo), TNF-β (*p* = 0.03)	IL-8 (p = 0.02), IL-15 (p = 0.00)
Suhadolnik, et al. [33]	IFN-α	NS	NS
Tomoda, et al. [34]	IL-1β, IL-4, IL-6, IL-10, IL-18, TGF-β, TNF- α	NS	TGF-β1

*HC* healthy controls, *IL* interleukin, *IFN* interferon, *LT* lymphotoxin, *TNF* tumor necrosis factor, *FGF* fibroblast growth factor, *VEGF* vascular endothelial growth factor, *G-CSF* granulocyte colony stimulating factor, *GM-CSF* granulocyte-macrophage colony stimulating factor, *CXCL* C-X-C motif ligand, *CCL* C-C motif ligand, *TGF* transforming growth factor, *SCF* stem cell factor, *NR p*-value not reported, *NS* not significant

^a^ Cytokines listed as assayed for a particular study that do not appear as either increased or decreased indicate their changes between the groups were not significant

This table contains the results of the studies pertaining to serum cytokine levels

**Table 3:** Study results for the secondary outcome of cytokine levels in other physiological fluids

		Cytokine Levels	
Author	Cytokines Analysed	Increased in CFS/ME vs. HC (p-value)^a^	Decreased in CFS/ME vs. HC (p-value)^a^
Cerebrospinal Fluid			
Hornig, et al. [22]	IL-2, IL-4, IL-5, IL-6, IL-7, IL-8, IL-10, IL-13, IL-15, IL-17A, IL-17F, GM-CSF, LIF, IL12p40, IL12p70, IFN-α2, IFN-β, IFN-γ, TNF-α, TNFβ, CCL2, CCL3, CCL4, CCL5, CCL7, CCL11, CXCL1, CXCL5, CXCL9, CXCL10, TGF-α, TGF-β, VEGF-A, FGF-basic, β-NGF, HGF, SCF, M-CSF, G-CSF, Resistin, VEGF-A, Leptin	CCL11 (0.0189), CXCL10 (0.0261)	IL-1RA (0.014), IL-1b (0.0003), IL-5 (0.0431), IL-6 (0.0074), IL-8 (0.033), IL-10 (0.0036), IL-12p40 (0.0026), IL-17F (0.0136), TNF-β (0.0071), SCF (0.001), M-CSF (0.0016), GM-CSF (0.034), G-CSF (0.0211), FGF-β (0.0278), VEGF-A (0.0138), LIF (< 0.0001), Resistin (0.0132)
Natelson, et al. [28]	IL-1α, IL-1β, IL-3, IL-4, IL-5, IL-6, IL-7, IL-8, IL-10, IL-12p40, IL-12p70, IL-13, IL-15, TNF-α, IFN-γ, GM-CSF, CCL5, CCL2	IL-8 (< 0.007), IL-10 (< 0.025)	none
Peterson, et al. [30]	IL-1β, IL-1RA, IL-2, IL-4, IL-6, IL-7, IL-8, IL- 9, IL-10, IL-12p70, IL-13, IL-15, IL-17, FGF-basic, CCL11, G-CSF, GM-CSF, IFN-5, CXCL10, CCL2, CCL3, CCL4, CCL5, TNF-α, VEGF	none	IL-10 (< 0.05)
Nasal Lavage			
Repka-Ramirez, et al. [31]	IL-8, TNF-α, NGF	NS	NS

*HC* healthy controls, *IL* interleukin, *IFN* interferon, LT lymphotoxin, *TNF* tumor necrosis factor, *FGF* fibroblast growth factor, *VEGF* vascular endothelial growth factor, *G-CSF* granulocyte colony stimulating factor, *GM-CSF* granulocyte-macrophage colony stimulating factor, *CXCL* C-X-C motif ligand, *CCL* C-C motif ligand, *TGF* transforming growth factor, *LIF* leukaemia inhibitory factor, *NGF* nerve growth factor, *HGF* hepatocyte growth factor, *SCF* stem cell factor, *M-CSF* macrophage colony stimulating factor, *NR p*-value not reported, *NS* not significant

^a^ Cytokines listed as assayed for a particular study that do not appear as either increased or decreased indicate their changes between the groups were not significant

This table contains the result of the studies pertaining to cytokine levels in other 263 physiological fluids

**Table 4:** Summary of cytokines analysed in the included studies

Cytokine	n	Reference(s)	Number of Studies indicating significant (*p* < 0.05) increases (‘n’, author(s))	Number of Studies indicating significant (p < 0.05) decreases (‘n’, author(s))
CCL2	6	[21, 22, 24, 25, 28, 30]	0	0
CCL3	5	[21, 22, 24, 25, 30]	0	0
CCL4	5	[21, 22, 24, 25, 30]	0	0
CCL5	5	[21, 24, 27, 29, 35]	1 [24]	0
CCL7	2	[21, 24]	0	0
CCL11	5	[21, 22, 24, 25, 30]	0	0
CCL13	1	[24]	0	0
CCL17	1	[24]	0	0
CCL19	1	[24]	1 [24]	0
CCL22	1	[24]	0	0
CCL24	1	[24]	1 [24]	0
CCL26	1	[24]	0	0
CXCL-1	2	[22, 25]	0	0
CXCL-5	2	[22, 25]	0	0
CXCL-9	3	[22, 24, 25]	0	1 [25]
CXCL-10	5	[21, 22, 24, 25, 30]	0	0
CX3CL-1	1	[24]	0	1 [24]
FGF	1	[21]	0	0
FGF-b	3	[22, 25, 30]	0	0
G-CSF	4	[21, 24, 29, 35]	0	0
GM-CSF	6	[21, 22, 24, 25, 28, 30]	0	0
HGF	2	[22, 25]	0	0
IFN-α	2	[25, 33]	0	0
IFN-α_2_	1	[21]	0	0
IFN-β	2	[22, 25]	0	0
IFN-γ	9	[20–22, 24, 25, 28–30, 32]	0	1 [28]
IL-1RA	3	[21, 25, 30]	0	0
IL-1α	5	[20, 24, 25, 28, 32]	1 [20]	0
IL-1β	9	[20, 21, 24–26, 28–30, 32]	2 [20, 28]	1 [25]
IL-2	7	[20–22, 24, 25, 30, 32]	0	0
IL-3	1	[28]	0	0
IL-4	9	[20–22, 24–26, 28, 30, 32]	1 [20]	0
IL-5	7	[20–22, 24, 25, 28, 32]	1 [20]	0
IL-6	11	[20–22, 24–30, 32]	2 [20, 27]	2 [21, 29]
IL-7	6	[21, 22, 24, 25, 28, 33]	1 [21]	1 [25]
IL-8	12	[20–22, 24–32]	2 [21, 29]	2 [20, 32]
IL-9	1	[30]	0	0
IL-10	10	[20–22, 24–26, 28–30, 32]	2 [26, 29]	0
IL-12p40	3	[22, 25, 28]	0	0
IL-12p70	8	[20–22, 24, 25, 28, 30, 32]	1 [20]	0
IL-12/23p40	1	[24]	0	0
IL-13	8	[20–22, 24, 25, 28, 30, 32]	0	1 [20]
IL-15	6	[20, 24, 25, 28, 30, 32]	0	1 [20]
IL-16	2	[22, 24]	0	1 [24]
IL-17	6	[19, 23, 24, 29, 31, 35]	0	0
IL-17A	1	[21]	0	1 [21]
IL-17F	2	[22, 25]	0	0
IL-23	2	[20, 32]	0	0
Leptin	2	[22, 25]	0	0
LIF	2	[22, 25]	0	0
LTα	2	[20, 32]	1 [20]	0
M-CSF	2	[22, 25]	0	0
NGF	1	[31]	0	0
β-NGF	1	[21]	0	0
Resistin	2	[22, 25]	0	1 [25]
SCF	2	[22, 25]	0	0
TNF-α	11	[20–22, 24–26, 28–32]	1 [29]	0
TNF-β	3	[22, 24, 25]	1 [32]	0
TGF-α	2	[22, 25]	0	0
TGF-β	3	[22, 25, 32]	1 [25]	1 [22]
TGF-β_1_	1	[23, 34]	1 [23]	1 [34]
VEGF	3	[21, 25, 30]	0	0
VEGF-A	2	[22, 24]	0	1 [24]

*IL* interleukin, *IFN* interferon, *LT* lymphotoxin, *TNF* tumor necrosis factor, *FGF* fibroblast growth factor, *VEGF* vascular endothelial growth factor, *G-CSF* granulocyte colony stimulating factor, *GM-CSF* granulocyte-macrophage colony stimulating factor, *CXCL* C-X-C motif ligand, *CCL* C-C motif ligand, *SCF* stem cell factor, *TGF* transforming growth factor

This table contains the frequency of the cytokines analysed in the included 269 studies, including a summary of the significant increases and decreases

### Quality assessment

Studies were evaluated for quality using the STROBE (strengthening the reporting of observational studies in epidemiology) checklist [35, 37] (Additional file 1: Table S1). Item one was altered because the stating of a study design in the title does not alter the overall quality of the paper.

## Results

**Fig. 1:**
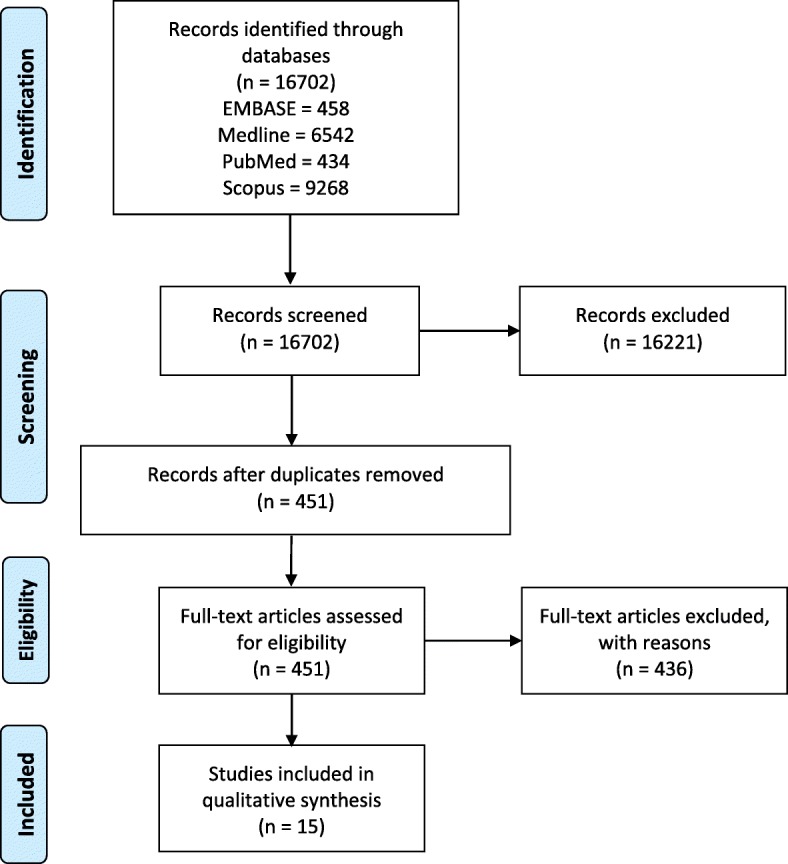
PRISMA flow diagram of literature search for included studies in this review of cytokines in CFS/ME/SEID

The results of the literature search are summarised by the PRISMA diagram in Fig. 1. A total of 16,702 records were identified from EMBASE (458), Medline (6542), PubMed (434) and Scopus (9268).

### Overview of studies

Figure 1 displays the PRISMA flow diagram with the number of studies that were included and excluded. The 15 studies included for review are summarised in Table 1. All studies in this review were observational case control studies that compared cytokines in CFS/ME/SEID patients to healthy controls.

### Quality assessment

All included publications were assessed for quality and bias by two authors using the STROBE checklist (provided in Additional file 1: Table S1). Each included the relevant information required to fulfil criteria, including: (i) title and abstract summary; (ii) background information; (iii) early outline of key study design; (iv) provided data sources and comparability measurements; (v) published summary of outcome measures; and (vi) provided an overall cautious interpretation of results. No publications commented on how the study size was arrived at or discussed the external validity of results. Fourteen of the 15 publications provided clear objectives of their studies [20–30, 32–34], clearly defined all variables [20–22, 24–34], and summarised key results [20, 21, 23–34]. Thirteen publications reported characteristics of study participants [20–27, 29, 31–34], and one publication explained their efforts to address any potential bias [25].

Each of the included publications provided an internationally accepted case definition for CFS/ME/SEID patients and stated the appropriate exclusion criteria. Minimal information was provided for the inclusion of healthy controls for the papers included in this systematic review.

### Study characteristics

Eleven studies reported serum cytokines [20, 21, 23–27, 29, 32–34], three reported CSF cytokines [22, 28, 30] and one reported cytokines from nasal lavage [31]. All of the included studies used one of two variations of cytokine detection and analysis. Eight used multiplex bead array assays (MBAA) [21, 22, 25–30] and seven used enzyme-linked immunosorbent assay (ELISA) [20, 23, 24, 31–34].

The frequency of the cytokines analysed in their included studies can be found in Table 4.

### Serum cytokines

There was a total of 64 cytokines analysed in the 15 studies. IL-8 was the most frequently analysed (*n* = 12). Regarding the studies that analysed serum cytokines (Table 2), there were statistically significant (*p* < 0.05) increases in 17 cytokines: CCL5 (*n* = 1) [21]; CCL19 (*n* = 1) [24]; CCL24 (*n* = 1) [24]; IL-1α (*n* = 1) [20]; IL-1β (*n* = 2) [20, 29]; IL-4 (*n* = 1) [20]; IL-5 (*n* = 1) [20]; IL-6 (*n* = 2) [20, 27]; IL-7 (*n* = 1) [21]; IL-8 (*n* = 2) [21, 29]; IL-10 (*n* = 2) [26, 29]; IL-12 (*n* = 1) [20]; LTα (*n* = 1) [20]; TGF-β (*n* = 1) [25]; TGF-β_1_ (*n* = 1) [23]; TNF-α (*n* = 1) [29] and TNF-β (*n* = 1) [32].

Statistically significant (p < 0.05) decreases were seen in 14 cytokines: CXCL-9 (*n* = 1) [24]; CX3Cl-1 (*n* = 1) [24]; IFN-γ (*n* = 1) [29]; IL-1β (*n* = 1) [24]; IL-6 (*n* = 2) [21, 29]; IL-7 (*n* = 1) [24]; IL-8 (*n* = 2) [20, 32]; IL-13 (*n* = 1) [20]; IL-15 (*n* = 1) [20]; IL-16 (*n* = 1) [24]; IL-17A (*n* = 1) [24]; Resistin (*n* = 1) [25]; TGF-β (*n* = 2) [24, 34] and VEGF-A (*n* = 1) [24]. The remainder were not significant.

Furthermore, it should be noted that one study [32] did not provide overall significance levels, and instead showed results of cytokine differences based upon age. For ages 18 to 50 years old, only IL-6 and IL-17 had statistically significant increases (*p* = 0.02). For ages over 50 years old, IL-4, IL-5 and IL-12p70 had a statistically significant increase (p = 0.02), and IL-15 had a statistically significant decrease (p = < 0.001). IL-8 and TNF-β were significantly decreased in both age groups (p = 0.02 and *p* = 0.01 respectively). Only the IL-8 and TNF-β results were included in the above overall analysis because it was statistically significant for adults of all ages included in the study.

### Cytokines in other physiological fluids

Studies that analysed cytokines in other physiological fluids are summarised in Table 3. In CSF, statistically significant increases were seen in four cytokines: CCL11 (*n* = 1) [22]; CXCL10 (*n* = 1) [22]; IL-8 (*n* = 1) [28] and IL-10 (*n* = 1) [28]. Statistically significant decreases were seen in: FGF-β (*n* = 1) [22]; G-CSF (*n* = 1) [22]; GM-CSF (*n* = 1) [22]; IL-1RA (*n* = 1) [22]; IL-1β (*n* = 1) [22]; IL-5 (*n* = 1) [22]; IL-6 (*n* = 1) [22]; IL-8 (*n* = 1) [22]; IL-10 (*n* = 1) [22]; IL-12p40 (*n* = 1) [22]; IL-17F (*n* = 1) [22]; LIF (*n* = 1) [22]; M-CSF (*n* = 1) [22]; Resistin (*n* = 1) [22]; SCF (*n* = 1) [22]; TNF-β (*n* = 1) [22] and VEGF-A (*n* = 1) [22]. There were no statistically significant differences between the cytokines present in nasal lavage in CFS/ME/SEID patients compared with healthy controls [31].

## Discussion

This systematic review has summarised the evidence currently available on cytokine levels in CFS/ME/SEID patients compared with healthy controls. These included serum cytokines, and cytokines from CSF and nasal lavage. Overall, 64 cytokines were analysed in the 15 studies selected, which were all observational case control studies of overall moderate quality. This study demonstrates the lack of evidence in alterations of cytokine levels between CFS/ME/SEID patients and healthy controls, and therefore, suggesting there is minimal potential for cytokines to be used as a reliable biomarker in CFS/ME/SEID.

### Study characteristics

Most of the studies in this review used the Fukuda (1994) criteria [8] for defining their patient population, however, this makes it difficult to identify a homogenous sample due to the broad, non-specific nature of the criteria [3, 38]. Other case definitions used in the identified studies were the CCC and the ICC, which compared with Fukuda, employ a more strict set of criteria and account for more diverse symptomatology in CFS/ME/SEID patients respectively [9, 39]. Limitations in these criteria are also comparable [3, 40].

Two main methods of cytokine analysis (MBAA and ELISA) from a variety of manufacturers were used in all of the studies identified. Traditionally, ELISA has been the standard for quantitative analysis of cytokines, although it is not as well suited for high output multiplex analyses compared with MBAA [41]. It should be noted that while both MBAA and ELISA are acceptable methods for cytokine analysis, comparing data from the two methods is difficult unless the same antibodies and similar reagents are used [41]. Furthermore, comparison of data between different MBAA assays is also limited by similar factors [41]. A consistent method of cytokine analysis is recommended for any future studies in this area to ensure comparability of data.

The use of strict exclusion criteria in our study to select for studies that removed any CFS/ME/SEID cases that were combined with other conditions minimised the risk that comorbidities played a role in the results found. Furthermore, any studies that induced cytokine expression in vitro were excluded, as the aim of this paper was not to examine the changes in immune cell cytokine expression. However, as cytokine expression is partially dependent on the function of immune cells, further research into this area would be warranted.

### Cytokine levels

CFS/ME/SEID is a complex condition with unknown aetiology that is manifested by multisystem involvement, including the cardiovascular, gastrointestinal, immune, metabolic and neurological systems [2]. The findings of this study indicate that of the 64 cytokines analysed, none appear to differ with any consistency between CFS/ME/SEID patients and healthy controls, in either serum or other physiological fluids, despite comparability between each study’s analytical methods. Nor does there appear to be any consistency between the study populations regarding pro-inflammatory and anti-inflammatory cytokines. The overall range of cytokines analysed in each of the studies also demonstrates that a wide approach to determining whether they play a key role in the aetiology of CFS/ME/SEID has been explored.

Included studies also attempted to explain inconsistencies between results regarding cytokine levels, with one study using two control groups, healthy controls and depression controls [33]; another examining and finding that cytokine levels were altered at night [26], and another suggesting that cytokines may vary with age [32]. Although many of the included studies age-matched their participants, one study analysed cytokines in middle-aged women [20], stating that it was most representative of the whole as 80% of CFS/ME/SEID patients are female [20, 42]. Ultimately, the inability to explain the inconsistencies in the data demonstrates that there is currently no conclusive evidence linking cytokine expression in physiological fluids and CFS/ME/SEID. However, as cytokines do not exist in isolation, a network analysis may be more appropriate [15].

The level of evidence identified in this review was overall of moderate quality with quality assessment demonstrating consistency between several publications. Interestingly, three studies that lacked in their methodology all investigated cytokines in physiological fluids other than serum. It should be also be noted that small modifications that would be unlikely to alter results would drastically improve the quality of the assessed papers. Main limitations of the included studies were: no methods mentioned for eliminating bias; not describing the study setting or participants, lack of power calculations or stating study limitations, failing to declare research funding and, not denoting areas for improvement if subsequent research should be undertaken.

Consequently, the current available evidence for differences in cytokine levels between healthy individuals and CFS/ME/SEID patients is inconclusive, despite the use of consistent methodologies and the availability of moderate-to-high quality studies. Overall study quality and study data could be improved by addressing the above limitations.

### Meta-analysis

A traditional meta-analysis of the studies (*n* = 15) was attempted, however, was unable to be performed according to the standards outlined in the literature [43]. The authors attempted to analyse significance of each cytokine measured (*n* = 64), however, no significant data (*p* < 0.05) were identified and effect sizes were unable to be calculated (data not published). The interpretation of results for this systematic review relied on *p*-values provided as no other statistical methods were available for data interpretation. A limitation of this systematic review is that not all publications provided actual p-values. Rather, these publications only provided a cut off for significance at *p* < 0.05.

### Quality assessment

Quality assessment was consistent among several publications. Shortcomings were due to limited information on sources of confounding variables and potential bias. Selection bias may be a potential issue for some publications as limited information was provided regarding the recruitment of healthy controls. The selection criteria for CFS/ME/SEID was mostly consistent throughout all publications and all adhered to internationally accepted criteria. Analysis of serum cytokine levels was consistent, indicating reliability of results. No publication reported any major limitations. Item one of the STROBE checklist was altered because all of the included studies failed to mention study design in their title, and it was deemed that such a shortcoming would not alter overall result quality or significance. No other modifications to the criteria were made in order to prevent falsely elevating the study quality results.

## Conclusions

The aim of this study was to examine and then review the current available evidence on cytokine levels in CFS/ME/SEID to determine whether there are changes in the circulating levels compared with healthy individuals. As cytokines have been thought to play a role in the aetiology of CFS/ME/SEID, their analysis may have helped lead to the development of a diagnostic test and/or targeted treatments. However, despite the consistent data, the findings of this review are inconclusive as to whether cytokines play any definitive role in CFS/ME/SEID, other than provide some evidence of a concurrent inflammatory process. Therefore, in light of these results, it is recommended that the aforementioned limitations of the studies be addressed, and a standardised protocol, including consistent use of case definitions and selection of homogenous CFS/ME/SEID populations that can be stratified, be developed for further evaluation into determining whether cytokines play a role in the aetiology of CFS/ME/SEID. Another recommendation includes further analysis within CFS/ME/SEID cohorts, due to the interconnection between pro-inflammatory and anti-inflammatory cytokines. Despite these results, progress toward a diagnostic test and targeted treatment(s) should continue in a variety of research domains.

Future research could also be targeted at immune cell function, and whether alterations in immune cell function play a role in the expression of cytokines, either in serum or other physiological fluids.

## Additional file

Additional file 1: **Table S1.** Summary of STROBE quality assessment for studies included in this systematic review. This table contains the combined results of the primary and secondary quality assessments undertaken for the studies included in this review. (DOCX 24 kb)

## Data Availability

All data generated or analysed during this study are included in this published article.

## Abbreviations

CCC: Canadian Consensus Criteria
CCL: C-C motif ligand
CFS: Chronic Fatigue Syndrome
CSF: Cerebrospinal Fluid
CXCL: C-X-C motif ligand
ELISA: Enzyme-linked immunosorbent assay
FGF: Fibroblast growth factor
G-CSF: Granulocyte colony stimulating factor
GM-CSF: Granulocyte-macrophage colony stimulating factor
HC: Healthy controls
HGF: hepatocyte growth factor
ICC: International Consensus Criteria
IFN: Interferon
IL: Interleukin
LIF: Leukaemia inhibitory factor
LT: Lymphotoxin
MBAA: Multiplex bead array assay
M-CSF: Macrophage colony stimulating factor
ME: Myalgic Encephalomyelitis
MeSH: Medical subject heading
NGF: Nerve growth factor
NR: Not reported (*p*-value)
NS: Not significant
PRISMA: Preferred Reporting Items for Systematic Reviews and Meta-Analyses
SCF: Stem cell factor
SEID: Systemic Exertion Intolerance Disease
STROBE: Strengthening the reporting of observational studies in epidemiology
TGF: Transforming growth factor
TNF: Tumor necrosis factor
VEGF: Vascular endothelial growth factor
