# Supplementary material for: A systematic review of cytokines in chronic fatigue syndrome/myalgic encephalomyelitis/systemic exertion intolerance disease (CFS/ME/SEID)
Source: BMC Neurol. 2019 Aug 24;19:207. doi: 10.1186/s12883-019-1433-0 (PMC6708220; doi:10.1186/s12883-019-1433-0)
Supplement: Supplementary file 1 — Table S1. Summary of STROBE quality assessment for studies included in this systematic review. This table contains the combined results of the primary and secondary quality assessments undertaken for the studies included in this review. (DOCX 24 kb) [file 12883_2019_1433_MOESM1_ESM.docx]

**Additional file 1: Table S1: Summary of STROBE quality assessment for studies included in this systematic review.**

|  |  | Introduction | | Methods | | | | | | | | | Results | | | | | Discussion | | | |  |
| --- | --- | --- | --- | --- | --- | --- | --- | --- | --- | --- | --- | --- | --- | --- | --- | --- | --- | --- | --- | --- | --- | --- |
| Author | **Title and Abstract^1^** | **Background and rationale^2^** | **Objectives^3^** | **Study Design^4^** | **Setting^5^** | **Participants^6^** | **Variables^7^** | **Data Sources and measurement^8^** | **Bias^9^** | **Study Size^10^** | **Quantitative Variables^11^** | **Statistical Methods^12^** | **Participants^13^** | **Descriptive Data^14^** | **Outcome Data^15^** | **Main Results^16^** | **Other Analyses^17^** | **Key Results^18^** | **Limitations^19^** | **Interpretations^20^** | **Generalisability^21^** | **Funding^22^** |
| Fletcher, *et al.* (2009) | 1 | 1 | 1 | 1 | 0 | 1 | 1 | 1 | 0 | 0 | 1 | 1 | 1 | 0 | 1 | 1 | 0 | 1 | 1 | 1 | 0 | 1 |
| Hardcastle, *et al.* (2015) | 1 | 1 | 1 | 1 | 0 | 1 | 1 | 1 | 0 | 0 | 1 | 1 | 1 | 1 | 1 | 1 | 0 | 1 | 0 | 1 | 0 | 1 |
| Hornig, *et al.* (2016) | 1 | 1 | 1 | 1 | 1 | 1 | 1 | 1 | 0 | 0 | 1 | 1 | 1 | 1 | 1 | 1 | 0 | 0 | 0 | 1 | 0 | 1 |
| Kennedy, *et al.* (2004) | 1 | 1 | 1 | 1 | 0 | 1 | 0 | 1 | 0 | 0 | 1 | 1 | 1 | 1 | 1 | 1 | 0 | 1 | 1 | 1 | 0 | 1 |
| Landi, *et al.* (2016) | 1 | 1 | 1 | 1 | 0 | 1 | 1 | 1 | 0 | 0 | 1 | 1 | 1 | 1 | 1 | 1 | 0 | 1 | 1 | 1 | 0 | 1 |
| Montoya J, *et al.* (2017) | 1 | 1 | 1 | 1 | 1 | 1 | 1 | 1 | 1 | 0 | 1 | 1 | 1 | 1 | 1 | 1 | 0 | 1 | 1 | 1 | 0 | 0 |
| Nakamura, *et al.* (2010) | 1 | 1 | 1 | 1 | 0 | 1 | 1 | 1 | 0 | 0 | 1 | 1 | 1 | 1 | 1 | 1 | 0 | 1 | 1 | 1 | 0 | 1 |
| Nas K, *et al.* (2011) | 1 | 1 | 1 | 1 | 0 | 1 | 1 | 1 | 0 | 0 | 1 | 1 | 1 | 1 | 1 | 1 | 0 | 1 | 0 | 1 | 0 | 0 |
| Natelson, *et al.* (2005) | 1 | 1 | 1 | 1 | 0 | 1 | 1 | 1 | 0 | 0 | 1 | 1 | 0 | 1 | 1 | 1 | 0 | 1 | 0 | 1 | 0 | 1 |
| Neu D, *et al.* (2014) | 1 | 1 | 1 | 1 | 1 | 1 | 1 | 1 | 0 | 0 | 1 | 1 | 1 | 1 | 1 | 1 | 0 | 1 | 0 | 1 | 0 | 0 |
| Peterson, *et al.* (2015) | 1 | 1 | 1 | 1 | 0 | 0 | 1 | 1 | 0 | 0 | 1 | 1 | 0 | 1 | 1 | 1 | 0 | 1 | 0 | 1 | 0 | 0 |
| Repka-Ramirez, *et al.* (2002) | 1 | 1 | 0 | 1 | 0 | 0 | 1 | 1 | 0 | 0 | 1 | 1 | 1 | 1 | 1 | 1 | 0 | 1 | 0 | 1 | 0 | 0 |
| Russell, *et al.* (2016) | 1 | 1 | 1 | 1 | 1 | 1 | 1 | 1 | 0 | 0 | 1 | 1 | 1 | 1 | 1 | 1 | 0 | 1 | 1 | 1 | 0 | 1 |
| Suhadolnik, *et al.* (2004) | 1 | 1 | 1 | 1 | 0 | 1 | 1 | 1 | 0 | 0 | 1 | 1 | 1 | 1 | 1 | 1 | 1 | 1 | 1 | 1 | 0 | 1 |
| Tomoda, *et al*. (2005) | 1 | 1 | 1 | 1 | 0 | 1 | 1 | 1 | 0 | 0 | 1 | 1 | 1 | 0 | 1 | 1 | 0 | 1 | 0 | 1 | 0 | 1 |

Criteria [22, 23]

^1^ A descriptive title (see N.B.), and; provide in the abstract an informative and balanced summary of what was done and what was found.

^2^ Explain the scientific background and rationale for the investigation being reported

^3^ State specific objectives, including any pre-specified hypotheses

^4^ Present key elements of study design early in the paper

^5^ Describe the setting, locations, and relevant dates, including periods of recruitment, exposure, follow-up, and data collection

^6^ Give the eligibility criteria, and the sources and methods of case ascertainment and control selection. Give the rationale for the choice of cases and controls

^7^ Clearly define all outcomes, exposures, predictors, potential confounders, and effect modifiers. Give diagnostic criteria, if applicable

^8^ For each variable of interest, give sources of data and details of methods of assessment (measurement). Describe comparability of assessment methods if there is more than one group

^9^ Describe any efforts to address potential sources of bias

^10^ Explain how the study size was arrived at

^11^ Explain how quantitative variables were handled in the analyses. If applicable, describe which groupings were chosen and why

^12^ Describe all statistical methods, including those used to control for confounding; Describe any methods used to examine subgroups and interactions; Explain how missing data were addressed; if applicable, explain how matching of cases and controls was addressed; describe any sensitivity analyses

^13^ Report numbers of individuals at each stage of study—eg numbers potentially eligible, examined for eligibility, confirmed eligible, included in the study, completing follow-up, and analysed; Give reasons for non-participation at each stage

^14^ Give characteristics of study participants (eg demographic, clinical, social) and information on exposures and potential confounders; Indicate number of participants with missing data for each variable of interest

^15^ Report numbers of outcome events or summary measures

^16^ Give unadjusted estimates and, if applicable, confounder-adjusted estimates and their precision (eg, 95% confidence interval). Make clear which confounders were adjusted for and why they were included; Report category boundaries when continuous variables were categorized; If relevant, consider translating estimates of relative risk into absolute risk for a meaningful time period

^17^ Report other analyses done—eg analyses of subgroups and interactions, and sensitivity analyses

^18^ Summarise key results with reference to study objectives

^19^ Discuss limitations of the study, taking into account sources of potential bias or imprecision and discuss both direction and magnitude of any potential bias

^20^ Give a cautious overall interpretation of results considering objectives, limitations, multiplicity of analyses, results from similar studies, and other relevant evidence

^21^ Discuss the generalisability (external validity) of the study results

^22^ Give the source of funding and the role of the funders for the present study and, if applicable, for the original study on which the present article is based

*If primary outcome measure not stated then key measure used

N.B. Item one was altered to include only a title (without study design) and provide in the abstract an informative and balanced summary of what was done and what was found.
